# Participation and performance trends in short-, medium, and long-distance duathlon

**DOI:** 10.1038/s41598-023-36050-2

**Published:** 2023-06-08

**Authors:** Jonas Turnwald, Caio Victor Sousa, Marilia Santos Andrade, Mabliny Thuany, Ivan Cuk, Pantelis Theodoros Nikolaidis, Katja Weiss, Beat Knechtle

**Affiliations:** 1 Institute of Primary Care, University of Zurich, Zurich, Switzerland; 2 Health and Human Sciences, Loyola Marymount University, Los Angeles, USA; 3 Department of Physiology, University of Sao Paulo, Sao Paulo, Brazil; 4 Faculty of Sports, University of Porto, Porto, Portugal; 5 Faculty of Sport and Physical Education, University of Belgrade, Belgrade, Serbia; 6 School of Health and Caring Sciences, University of West Attica, Athens, Greece; 7 Medbase St. Gallen Am Vadianplatz, Vadianstrasse 26, 9001 St. Gallen, Switzerland

**Keywords:** Environmental sciences, Environmental social sciences

## Abstract

Participation and performance trends of male and female athletes have been thoroughly analyzed in various endurance sports. Knowing these trends can help coaches and athletes prepare for competitions and may influence their training strategy and career planning. However, duathlon events—consisted of two splits of running (Run1 and Run2) interspersed by a split of cycling (Bike)—have not been thoroughly studied, unlike other endurance sports. The present study aimed to compare participation and performance trends in duathletes who competed in duathlon races hosted by World Triathlon or affiliated National Federations between 1990 and 2021. A total of 25,130 results of age group finishers who competed in run-bike-run duathlon races of varying distances were analyzed with different general linear models. Races were divided into three distances: short-distance (up to 5.5 km run, 21 km bike, 5 km run), medium-distance (5–10 km run, 30–42 km bike, 7–11 km run) and long-distance (at least 14 km run, 60 km bike, 25 km run). On average, women represented 45.6% of all finishers in short-distance, 39.6% in medium-distance and 24.9% in long-distance duathlon races. Throughout the years, men were consistently faster than women in all three race legs (Run 1, Bike, and Run 2) in all three distances across all age groups, and women could not reduce the performance gap. Concerning the age of peak performance, duathletes of the age group 30–34 finished most often in the top three in short- and medium-distance duathlons, whereas male duathletes of the age group 25–29 and female duathletes of the age group 30–34 finished most often in the top three in long-distance duathlons. Women participated less, especially in longer distances, and were constantly slower than men. Duathletes of the age group 30–34 finished most often in the top three. Future studies should analyze participation and performance trends in further subgroups (e.g., elite athletes) and pacing behaviours.

## Introduction

Non-professional endurance sports have been consistently growing in popularity during the last decades. Accordingly, the scientific community has studied participation and performance trends in various sports such as triathlon^1,2^, distance running^3–5^, cycling^6,7^ and duathlon^8–10^. Duathlon is a unique multi-discipline sport in which athletes compete in a run-bike-run format. It is internationally governed by Word Triathlon (WT), formerly the International Triathlon Union (ITU). WT distances include a sprint-distance (5 km run, 20 km bike, 2.5 km run), standard-distance (5–10 km run, 30–40 km bike, 5 km run), middle-distance (10–20 km run, 60–90 km bike, 10 km run) and long-distance (10–20 km run, 120–150 km bike, 20–30 km run), but individual race distances can vary^11^. Participation and performance trends in duathlons have been investigated before. Nonetheless, to the best of our knowledge, the current literature is either based on a specific race^8,10,12^ or a specific distance^9^.

The performance gap between sexes is one of the main points of interest in endurance sports research. Consistent with studies on other endurance sports^1,3–6,13^, slower race times of women were observed in previously studied duathlon events^8–10^. Recently, Romero-Ramos et al.^9^ analyzed performance differences, with regard to age and sex, of the top ten age group athletes competing in the ITU Duathlon World Championships from 2005 to 2016 on the standard-distance length. Men outperformed women in all age groups in all race legs and with advancing age, the differences between both sexes increased^9^. Previous investigations focused on the Powerman Zofingen with its two distances (short-distance: ~ 10 km run, 50 km bike, 5 km run; long-distance: ~ 10 km run, 150 km bike, 30 km run)^8,10^. A consistent sex difference of ~ 18–19% in all race legs and total times was observed in the annual top ten elite athletes who participated in the long-distance races between 2002 and 2011^10^. When all finishers of the short- and long-distance races from 2003 to 2017 were analyzed, the sex difference was similar in both versions (~ 8%)^8^. These differences in race times between females and males might be explained by physiological, anthropometric, genetic, hormonal and psychological factors^13–15^. However, the sex gap seems to be dependent on the race distance. In other endurance sports, such as distance running, the sex gap has been shown to decrease in ultra-endurance-distances^15–17^. In a study by Waldvogel et al.^16^, a higher sex gap was observed in 50 mile (9.13%) than in 100 mile (4.41%) ultra-marathon races.

Age is another important aspect affecting performance in endurance sports. With advancing age, cellular deterioration and loss of tissue function occur, affecting physical performance in different manners based on the specific requirements of the activity^18–20^. Compared to sprint races, the age of peak performance (APP) seems to be higher in endurance races^2,20^. This relationship is also reflected in endurance events of different distances. For instance, Nikolaidis et al.^12^ investigated the APP in the short- and long-distance races of the Powerman Zofingen and reported that the fastest age group was younger in the short-distance race (age group 20–24) than in the long-distance race (age group 25–29). Conversely, Romero-Ramos et al.^9^ reported a higher APP (age group 30–34) in both genders when the overall performance of the top ten athletes of each age group at the ITU Duathlon World Championships on the standard-distance was compared. As the race distances (~ 10 km run, 40 km bike, 5 km run) were shorter compared to the short version of the Powerman Zofingen (~ 10 km run, 50 km bike, 5 km run), the difference in the observed APP might be explained by the different study designs and the specific characteristics of the Powerman Zofingen. This highlights the importance of a more extensive dataset for a better understanding of the trends in duathlon^9,12^. Up to now, no study regarding the APP in the sprint-distance exists.

The two disciplines, running and cycling, represent different types of locomotion with their own anthropometric and physiological correlates^21,22^. When the age-related performance decline of each discipline was analyzed separately, the cycling performance could be better maintained than the running performance in older athletes^8,9,23^. This phenomenon was also observed in triathlon events, where the performance decline with increasing age was more prominent in swimming and running than in cycling^1^.

Little is known so far concerning participation trends in duathlon. When investigating finishers of the Powerman Zofingen from 2003 to 2017, 15.2% of all finishers in the long-distance and 15.9% of all finishers in the short-distance were women^8^. In the shorter ITU Duathlon World Championships (standard-distance), higher participation of women was observed from 2005 to 2016. Romero-Ramos et al.^9^ reported that 23.5% of all finishers were women. More studies have been conducted on triathlon races, with an increase in female participants observed since the 1980s^1^. Also, in triathlon, it seems that women tend to compete in shorter than longer distances^1,24–26^.

Although the above-mentioned literature provides some information about participation and performance trends in duathlon, no study has investigated the worldwide trends across different race distances so far. Knowledge of these trends would not only be interesting for scientists but could also help athletes and coaches prepare for races and could influence their training strategy depending on the sex and age of an athlete and the specific distance of a race. Furthermore, duathletes who are aware of different APPs in different race distances would be able to plan their career more precisely.

Therefore, the present study aimed to investigate the worldwide participation and performance trends in duathlon with an extensive dataset, including results from finishers who participated in duathlon races worldwide across different distances over several decades. Based upon the previously mentioned findings, we hypothesized firstly that more male than female finishers would be recorded for all distances and especially for longer distances, secondly, that men would be faster than women, thirdly that the sex gap would narrow throughout the years and fourthly, that the APP is higher in longer race distances.

## Methods

### Ethical approval and consent to participate

This study was approved by the Institutional Review Board of Kanton St. Gallen, Switzerland, with a waiver of the requirement for informed consent of the participants as the study involved the analysis of publicly available data (EKSG 01/06/2010). The study was conducted in accordance with recognized ethical standards according to the Declaration of Helsinki adopted in 1964 and revised in 2013.

### Duathlon events

Results of international events hosted by WT or affiliated National Federations were obtained from the results section of WT's official website^27^. To ensure comparability, we only included regular international duathlon races that were either World Championship or Continental Championship races, and excluded Cross- or Winter-Duathlons. A total of 187 races were identified, which have taken place from 1990 to 2021. However, distances were not stated on the downloadable result lists. Therefore, information about the race distances had to be retrieved in multiple ways. The race distances were listed in the “Program notes” section for some participant groups. If this was not available, we searched the event page of a specific race with the three tabs “Event Info”, “Local Info” and “Contact” for any information. If no distance was available, we scanned the event page for an external link to the official event website of the race. If available, we thoroughly browsed this website to find the relevant data. An external link was found for some races, but the website was no longer active or contained non-corresponding content. In this case, we searched for archived versions of the event website on the Wayback Machine of the Internet Archive to get any information regarding the distances^28^. Nonetheless, no distances could be retrieved for some events/participant groups. We only included data of participant groups, if a clear association of a respective participant group with a specific distance was present. As individual race distances differed and did not always match a specific WT distance, we divided the races into three distances: short-distance (up to 5.5 km run, 21 km bike, 5 km run), medium-distance (5–10 km run, 30–42 km bike, 7–11 km run) and long-distance (at least 14 km run, 60 km bike, 25 km run).

For the purpose of this study, we only included successful finishers of adult age group categories who competed in a duathlon race in a run-bike-run race mode. Except for the age group 18–19 years, each age group covers a five-year period (20–24 years through to 85–89 years). Required data from the race results included the name of an athlete, the sex, the split times, the total time and the age group. The year and name of an event, obtained from the corresponding event page on WT's website, and the distance were added. No races from the years 1990, 1992, 1996, 1997, and 2004 could be included. In short-distance duathlon, the first race that could be included was in 2011, in medium-distance duathlon in 1991, and in long-distance duathlon in 2002. Excluded were results from the 1997 Guernica ITU Duathlon World Championships, as the split times did not match the overall times in most cases, and the 2003 Affoltern ITU Duathlon World Championships, as the stated distance appeared to be wrong. Moreover, finishers with empty race times and statistical outliers in any of the race legs (slower/faster by three standard deviations from the mean) were excluded. In total, 66 races met the inclusion criteria.

### Statistical analysis

Descriptive statistics were presented using mean ± standard deviation and frequencies. All data showed parametric distribution and homogeneity of variance through the Kolmogorov–Smirnov's and Levene's tests, respectively. Average speed (kilometers per hour (km/h)) was established as the dependent variable for all models. General linear models (GLM) with two factors (two-way ANOVA) were applied for each distance (short, medium, and long) considering the independent factors “sex × age group” and “sex × calendar year”. Further GLM were conducted for men and women separately with “event distance × age group” as independent factors. Fisher's least significant difference was applied as a post-hoc test to identify specific differences between independent factors. Partial eta square (η_p_^2^) was applied as a measure of effect size, considering η_p_^2^ = 0.01 as a small effect, η_p_^2^ = 0.06 as a moderate effect, and η_p_^2^ = 0.14 as a large effect. Statistical significance was defined as *p* < 0.05. All statistical analyses were carried out with Statistical Software for the Social Sciences (IBM^®^ SPSS v.25, Chicago, Ill, USA).

## Results

**Figure 1:**
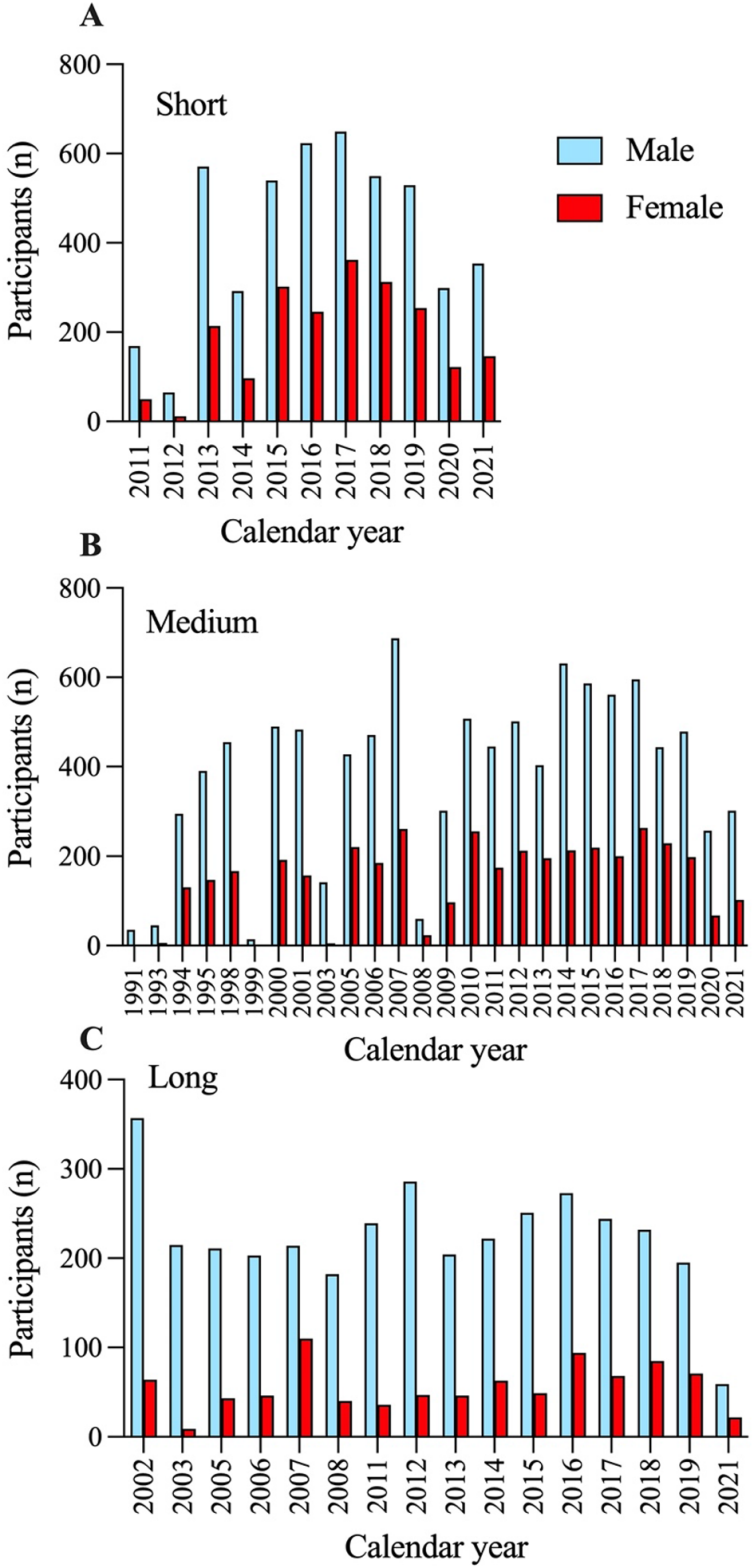
Participation of men and women in short- (**A**), medium- (**B**), and long-distance (**C**) duathlon across calendar years.

A total of 25,130 finishers were included. Short-distance duathlon included 4641 men and 2118 women (n = 6759), medium-distance duathlon included 9970 men and 3921 women (n = 13,891), and long-distance duathlon included 3587 men and 893 women (n = 4480). Women's participation in individual races ranged from 18.5 to 55.9% in relation to men in short-distance duathlon (average: 45.6%), 13.3–51.7% in medium-distance duathlon (average: 39.6%) and 4.2–37.3% in long-distance duathlon (average: 24.9%). See Fig. 1 for detailed participation by sex and year.

Performance trends across age groups showed significant effects of both sex and age group for short-, medium-, and long-distance duathlon across all three race legs of the duathlon race (Run 1, Bike, Run 2). See Table 1 for details.

**Table 1:** General linear model results with average speed as the dependent variable.

Duathlon		Sex			Age group			Sex × Age group		
		F	*p*	η_p_^2^	F	*p*	η_p_^2^	F	*p*	η_p_^2^
Short	Run 1	180.6	< 0.001	0.66	58.2	< 0.001	0.99	5.0	< 0.001	0.01
	Bike	147.2	< 0.001	0.20	68.2	< 0.001	0.99	1.5	0.14	< 0.01
	Run 2	135.4	< 0.001	0.47	58.3	< 0.001	0.99	3.5	< 0.001	0.01
Medium	Run 1	231.0	< 0.001	0.38	108.7	< 0.001	0.99	4.3	< 0.001	0.01
	Bike	104.7	< 0.001	0.04	71.0	< 0.001	0.99	1.4	0.16	< 0.01
	Run 2	137.3	< 0.001	0.10	156.4	< 0.001	0.99	2.0	0.02	< 0.01
Long	Run 1	119.8	< 0.001	0.30	31.7	< 0.001	0.97	1.4	0.16	< 0.01
	Bike	119.9	< 0.001	0.29	19.4	< 0.001	0.95	1.4	0.19	< 0.01
	Run 2	41.6	< 0.001	0.10	23.0	< 0.001	0.95	1.15	0.32	< 0.01

**Figure 2:**
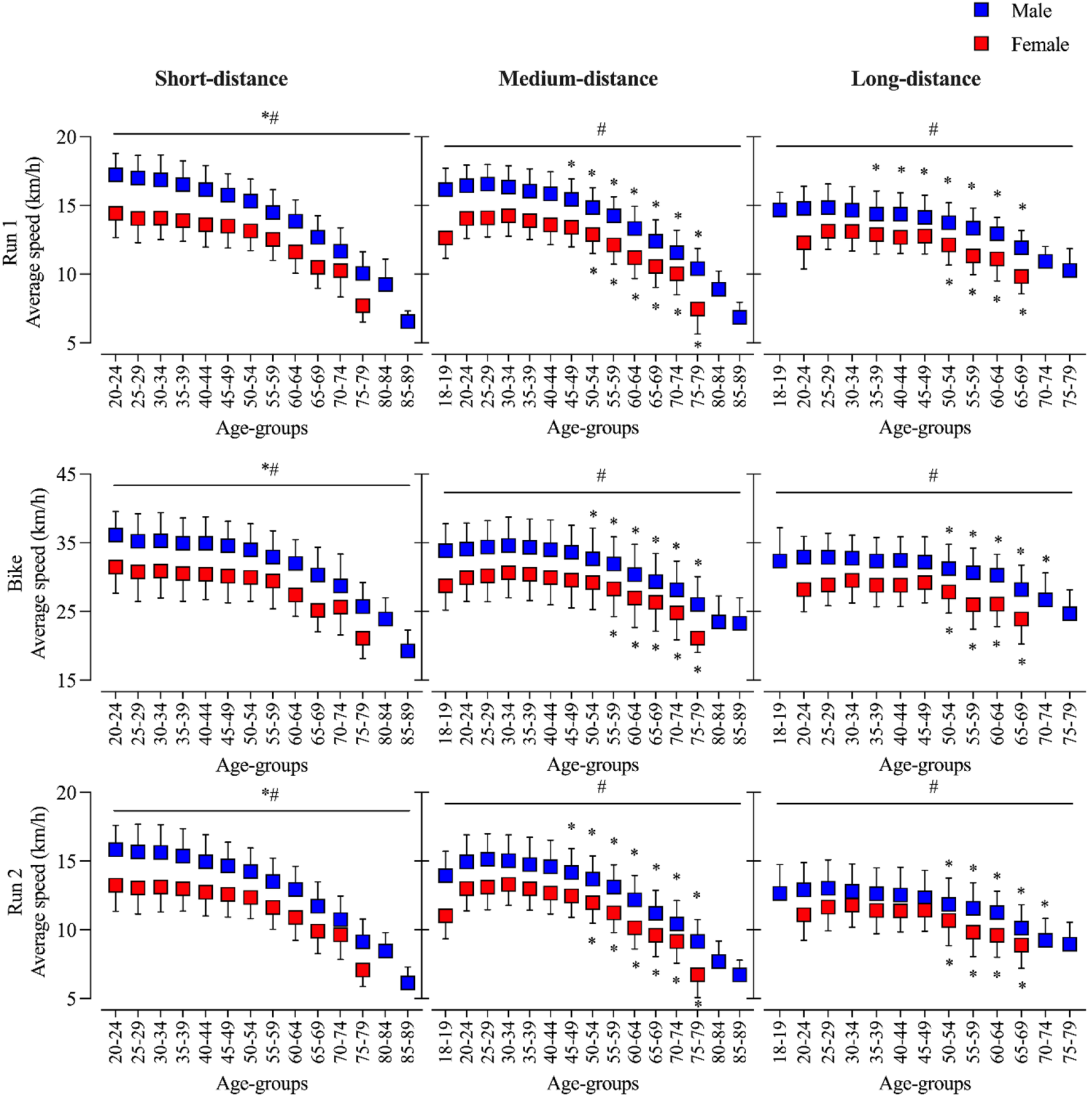
Average speed in the three duathlon race legs of men and women in short-, medium-, and long-distance duathlon across age groups. * over line: statistical significance between all age groups; *: statistical significance in comparison to the previous age group; # over line: statistical significance for sex across all age groups.

Pairwise comparisons showed that men had better performances than women across all age groups in all three race legs (Run 1, Bike, and Run 2) in all three duathlon distances. Finally, age group pairwise comparisons showed that, in short-distance duathlon, the age group was always significantly different from the previous one, but in medium-distance duathlon, the run performance started to drop at the age group 45–49 years in men and age group 50–54 years in women, whereas the bike performance started to drop at the age group 50–54 years in men and age group 55–59 years in women. In long-distance duathlon, the first running leg was stable until the age group 30–34 years in men and age group 50–54 years in women, whereas the bike performance and the second running leg were stable until the age group 50–54 years in both men and women. See Fig. 2.

Performance trends across calendar years showed significant effects of both sex and calendar years for short-, medium-, and long-distance duathlon across all three race legs of the duathlon race (Run 1, Bike, Run 2). See Table 2 for details.

**Table 2:** General linear model results with average speed as the dependent variable.

Duathlon		Sex			Calendar year			Sex × Calendar year		
		F	*p*	η_p_^2^	F	*p*	η_p_^2^	F	*p*	η_p_^2^
Short	Run 1	275.2	< 0.001	0.94	8.6	0.001	0.90	3.7	< 0.001	0.01
	Bike	292.7	< 0.001	0.94	43.7	< 0.001	0.98	3.9	< 0.001	0.01
	Run 2	230.5	< 0.001	0.93	10.4	< 0.001	0.91	3.3	< 0.001	0.01
Medium	Run 1	303.8	< 0.001	0.32	31.6	< 0.001	0.97	2.0	< 0.001	< 0.01
	Bike	245.1	< 0.001	0.31	98.0	< 0.001	0.99	2.2	0.001	< 0.01
	Run 2	177.7	< 0.001	0.24	25.0	< 0.001	0.96	2.1	0.001	< 0.01
Long	Run 1	870.6	< 0.001	0.96	70.9	< 0.001	0.98	0.56	0.91	< 0.01
	Bike	714.4	< 0.001	0.96	65.2	< 0.001	0.99	0.78	0.19	0.99
	Run 2	226.9	< 0.001	0.88	27.6	< 0.001	0.97	0.75	0.77	< 0.01

**Figure 3:**
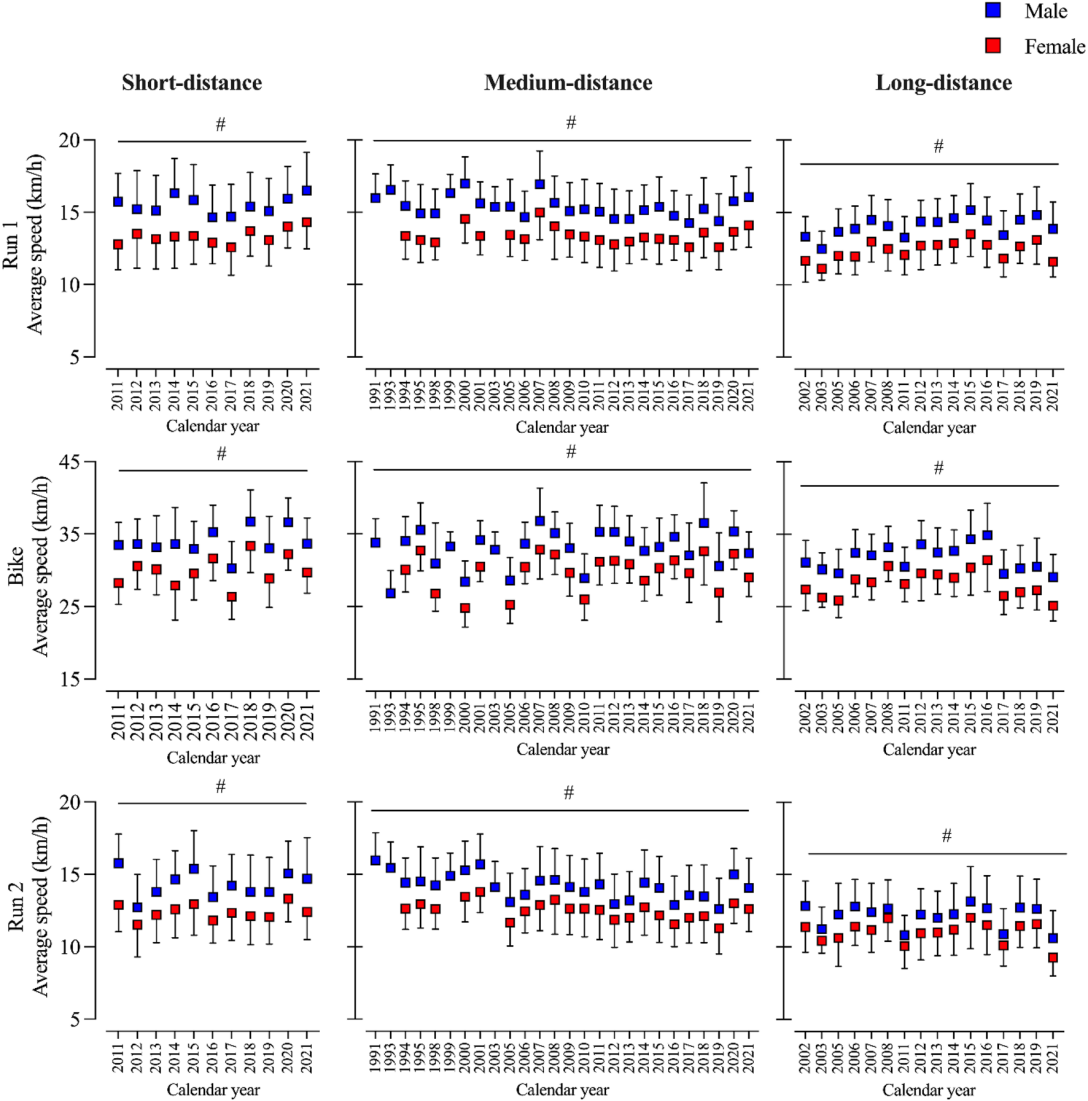
Average speed in the three race legs of men and women in short-, medium-, and long-distance duathlon across calendar years. # over line: statistical significance for sex across all calendar years.

Pairwise comparisons showed that men were consistently faster than women in all race legs and distances across all calendar years and no trend was observed that women reduced the sex gap throughout the years. Additionally, no apparent performance trend was seen in any distance throughout the years. See Fig. 3.

**Figure 4:**
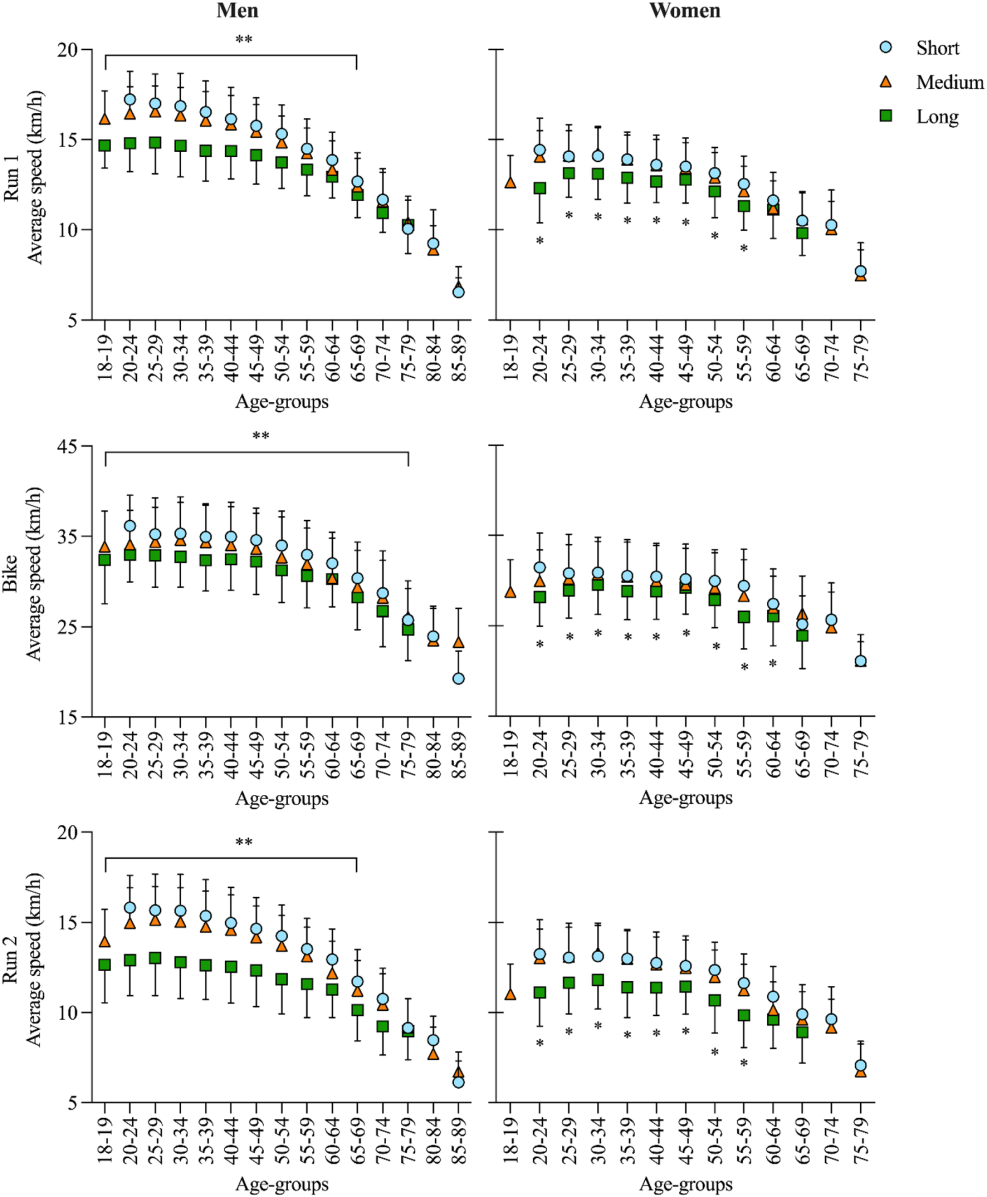
Average speed in the three race legs of men and women separately in short-, medium-, and long-distance duathlon across age groups. ** over line: statistical significance between all three events; *: statistically significant from the other two events.

The GLM for men showed significant “event” and “age group” effects for Run 1 (event: F = 39.5, *p* < 0.001, η_p_^2^ = 0.52; age group: F = 76.9, *p* < 0.001, η_p_^2^ = 0.98; interaction: F = 9.5, *p* < 0.001, η_p_^2^ = 0.01), Bike (event: F = 24.5, *p* < 0.001, η_p_^2^ = 0.14; age group: F = 82.9, *p* < 0.001, η_p_^2^ = 0.96; interaction: F = 2.6, *p* < 0.001, η_p_^2^ < 0.01) and Run 2 (event: F = 64.2, *p* < 0.001, η_p_^2^ = 0.54; age group: F = 75.2, *p* < 0.001, η_p_^2^ = 0.97; interaction: F = 6.0, *p* < 0.001, η_p_^2^ = 0.01). Similar results were found in women for Run 1 (event: F = 22.7, *p* < 0.001, η_p_^2^ = 0.18; age group: F = 65.7, *p* < 0.001, η_p_^2^ = 0.96; interaction: F = 2.0, *p* = 0.004, η_p_^2^ = 0.01), Bike (event: F = 13.8, *p* < 0.001, η_p_^2^ = 0.11; age group: F = 21.1, *p* < 0.001, η_p_^2^ = 0.88; interaction: F = 1.8, *p* = 0.016, η_p_^2^ = 0.01) and Run 2 (event: F = 38.9, *p* < 0.001, η_p_^2^ = 0.32; age group: F = 48.7, *p* < 0.001, η_p_^2^ = 0.95; interaction: F = 2.1, *p* = 0.002, η_p_^2^ = 0.01). See Fig. 4 for details.

Pairwise comparisons for the men models showed that the average speeds in the three race distances differed from each other up to the age group 65–69 years in the running legs and age group 70–74 years in the cycling leg. For the women models, a slower average speed was observed in long-distance races in comparison to the other distances up to the age group 55–59 years in the running legs and age group 60–64 years in the cycling leg (Fig. 4).

**Figure 5:**
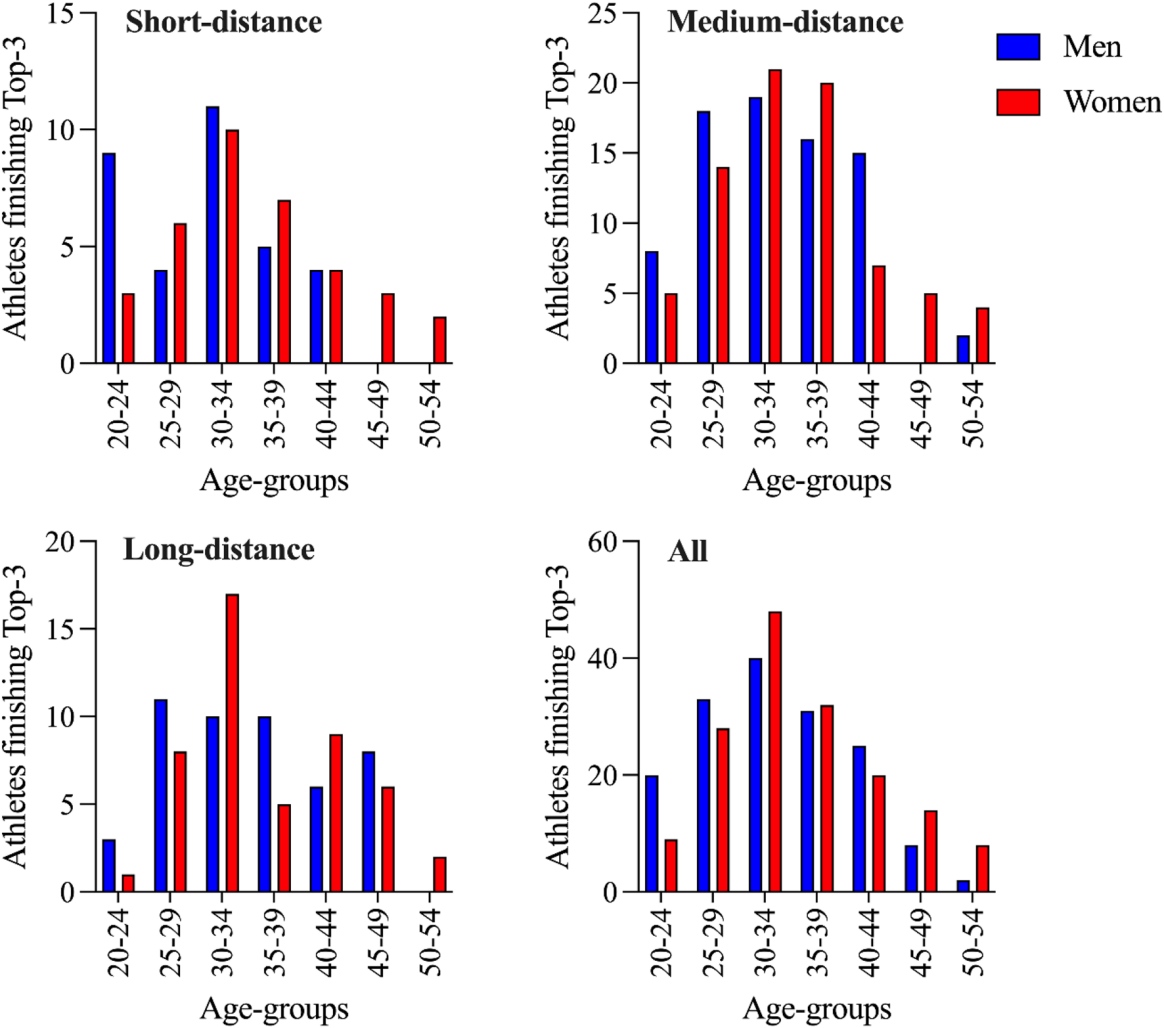
The number of athletes in each age group who finished in the analyzed races in the top three by sex and distance.

In the analyzed short- and medium-distance races, male and female athletes of the age group 30–34 years finished most often in the top three compared to other age groups. In long-distance races, men of the age group 25–29 years and women of the age group 30–34 years finished most often in the top three. Overall, when all distances were considered, the age group 30–34 years was the most prevalent one in the top three in men and women. See Fig. 5 for details.

## Discussion

This study intended to investigate the worldwide participation and performance trends of short-, medium- and long-distance duathlon over several decades. The participation in the investigated races did not increase over the years. This finding might not be generalized to the sport itself, as we considered only events hosted by WT or affiliated National Federations which were listed on WT's website and did not compare the same races every year. Furthermore, the present study analyzed world and continental championships, but not local events, which may be preferred by age group athletes. Non-elite athletes often face real-world commitments and financial constraints that can make it difficult for them to travel to events and compete^29^. In other endurance sports, such as long-distance running and cycling, an increased participation rate was observed in recent years^30–32^. In addition, the number of members in national federations has grown in the last decades. In Germany, for example, the number of members of the National Triathlon Federation has more than doubled between 2001 and 2022^33,34^.

An important finding was the lower number of female duathletes compared to male duathletes in all distances across all years. Women accounted on average for 45.6% of finishers in short-distance duathlon, 39.4% in medium-distance duathlon and 24.9% in long-distance duathlon. The lower rate of women finishers in longer race distances is in accordance with previous findings in triathlon and might be explained by motivational reasons, differences in training behaviour and sociocultural conditions^1,35,36^.

Regarding performance, men were faster than women in all race legs in all distances across all age groups and calendar years. There is extensive literature on factors that explain the differences in performance between men and women in endurance sports^13–15,36^. In addition to physiological differences, such as the lower maximal oxygen uptake (VO_2_max) in female athletes, morphological differences, social factors, psychological factors and differences in training characteristics have to be considered concerning the sex gap^13,37^. Female athletes were able to reduce the sex gap in ultra-endurance sports throughout the years, for example, in most ultra-marathon distances^38^ and ultra-cycling distances^7^. However, our hypothesis was not confirmed, as we did not find such a trend in any of the investigated distances throughout the years. Based on past findings, the analyzed distances in this study were not long enough to see such a trend, as the physiological and morphological advantages of women (e.g., better fatigue resistance, greater substrate efficiency and lesser energetic demands) rather seem to play a role in ultra- and extreme distances^15^.

Moreover, no apparent performance trend could be observed in any of the investigated distances throughout the years. While Nikolaidis et al.^8^ also found an unchanged performance of male and female finishers in the “Powerman Zofingen” from 2003 to 2017, Gallman et al.^39^ found an increased performance of the annual top ten male and female triathletes who competed at the Ironman Hawaii from 1983 to 2012. Methodological differences, including sample size, time frame and statistical procedures, may be related to the differences in these findings. For example, Gallman et al.^39^ analyzed the results of the top ten elite athletes, whereas we analyzed all successful finishers of adult age group categories. In many studies on performance trends in marathon races, a phenomenon was observed that “the faster get faster and the slower get slower”^40–43^. It is important to note that we analyzed data from multiple races, with differences in drafting rules, weather conditions, and track specifications, and not one specific race over a period of time, what may have impacted the results.

Another finding was that in short-distance duathlon, a statistically significant decline in performance with increasing age groups could be observed from the first age group (20–24 years), whereas the performance in medium- and long-distance duathlon was relatively stable up to a specific age group. In the analyzed medium-distance races, a statistically significant drop in performance in the first and second run was for the first time observed at the age group 45–49 years in men and 50–54 years in women, while the cycling performance dropped later at the age group 50–54 years in men and 55–59 years in women. Previous studies on multi-discipline sports already showed that the age-related performance decline seems to be higher in running than in cycling^1,19,44,45^. This might be related to the distinct characteristics of the two disciplines. Running is a weight-bearing stretch–shortening activity with a predominantly eccentric type of muscle action compared to cycling, which is a non-weight-bearing activity with concentric contractions^46,47^. However, it is noteworthy that in a study performed by Swinnen et al.^48^ running-specific training could improve running economy while the cycling economy could not be improved by cycling-specific training. Regarding long-distance duathlon, a statistically significant drop in performance in the first run was for the first time observed at the age group 30–34 years in men and 50–54 years in women, while in the cycling and second running leg performance dropped at the age group 50–54 years in men and women.

A reason that the performance in medium- and long-distance duathlon was relatively stable up to a specific age group in contrast to short-distance duathlon might be that generally, more experienced athletes compete in longer race distances and it was postulated before that the amount of experience is highly important for the performance in multi-discipline sports^49^.

When we analyzed the average speeds of men and women separately in the three race distances across the different age groups, particular differences could be observed. In men, a statistically significant difference between the average speeds of the three race distances could be observed up to the age group 65–69 years in both running legs and 70–74 years in the cycling leg. In women, on the other hand, only the average speed in long-distance duathlon was statistically significantly slower compared to the other two distances up to the age group 55–59 years in both running legs and 60–64 years in the cycling leg. Interestingly, no statistically significant difference could be observed between the average speeds of women in short- and medium-distance races. Although this phenomenon does not make physiological sense, it indicates that women have a lot of room for performance improvement in short-distance duathlon. In many endurance sports, it was previously shown that women adopted a more conservative pacing strategy than men^50–53^. This might be explained by differences regarding confidence, decision-making, risk perception and willingness^53^. For example, compared to men, women showed relatively lower speeds in the beginning and relatively higher speeds at the end of a 100 km ultra-marathon race^54^. One explanation could be that women did not allocate their energy resources in the best suitable manner. More studies are necessary to confirm or refute these results.

The only knowledge we have so far regarding pacing in a duathlon is derived from studies by Nikolaidis et al., who analyzed the effect of aging^23^, sex and performance level^50^ as well as the combined effect of aging and performance level^55^ on pacing. However, these studies are based solely on the “Powerman Zofingen” results from 2003 to 2017 with its two distances (10 km run, 50 km bike, 5 km run; ~ 10 km run, 150 km bike, 30 km run). The authors reported that women adopted a steadier pace and were relatively faster in the second run^50^. To the best of our knowledge, no information regarding pacing behaviours in short-distance duathlon exists.

Regarding the APP, male and female athletes of the age group 30–34 years finished most often in the top three in short- and medium-distance races. This confirms past findings by Romero-Ramos et al.^9^ who analyzed the performance of the top ten athletes of each age group who competed at the ITU Duathlon World Championships from 2005 to 2016 and found that athletes of the age group 30–34 years performed best in the standard-distance (~ 10 km run, 40 km bike, 5 km run). In long-distance duathlon, men of the age group 25–29 years and women of the age group 30–34 years finished most often in the top three in our study. Therefore, our hypothesis that the APP is higher in longer race distances could not be confirmed. This is in contrast to a study by Knechtle et al.^2^, who analyzed the different APPs of world-class triathletes in different race distances. The authors reported that men achieved the best performance at 27.1 ± 4.9 years in the Olympic distance, 28.0 ± 3.8 years in the Half-Ironman distance and 35.1 ± 3.6 years in the Ironman distance, while women were best at 26.6 ± 4.4, 31.6 ± 3.4 and 34.4 ± 4.4 years respectively. However, besides the differences regarding the modes of locomotion, the methodological approach was different and we were only able to determine the age group of the finishers and not their exact age.

### Limitations, strengths, and implications for future research

A limitation of this study is the use of secondary data. We were not able to consider important factors related to endurance performance in athletes of different competitive levels, such as anthropometric and physiological variables, training status, previous experience, drafting rules, technical equipment, track specifications and weather conditions. Due to the use of secondary data, we cannot exclude that some distances have been rounded. Moreover, data was missing in certain years. Other methodological designs, such as longitudinal studies, could offer more information about the effect of aging on duathlon performance. Nevertheless, this is the first study that investigated worldwide participation and performance trends in duathlon with results from finishers who participated in duathlon races worldwide across three different distances over several decades. Future studies should collect data about the above-mentioned variables and analyze participation and performance trends in further subgroups (e.g., elite athletes) as well as pacing behaviours in short-distance duathlon and the association between place of competition, participation and performance trends.

## Conclusion

More men than women competed in all distances and especially in longer distances. Men were generally faster across all age groups and no trend regarding the sex gap was observed at any distance throughout the years. The APP did not increase with an increase in the race distance. Men and women of the age group 30–34 finished most often in the top three in short- and medium-distance races, whereas in long-distance races, men of the age group 25–29 and women of the age group 30–34 finished most often in the top three.

## Data Availability

For this study, we have included official results and split times from the official website of WT https://triathlon.org. The datasets used and/or analyzed during the current study are available from the corresponding author on reasonable request.

## Abbreviations

APP: Age of peak performance
GLM: General linear models
ITU: International Triathlon Union
WT: World Triathlon

## References

[1] Lepers R, Knechtle B, Stapley PJ (2013). Trends in triathlon performance: Effects of sex and age. Sports Med..

[2] Knechtle R, Rüst CA, Rosemann T, Knechtle B (2014). The best triathletes are older in longer race distances: A comparison between Olympic, Half-Ironman and Ironman distance triathlon. Springerplus.

[3] Hanley B (2016). Pacing, packing and sex-based differences in Olympic and IAAF World Championship marathons. J. Sports Sci..

[4] Nikolaidis PT, Onywera VO, Knechtle B (2017). Running performance, nationality, sex, and age in the 10-km, Half-Marathon, Marathon, and the 100-km Ultramarathon IAAF 1999–2015. J. Strength Cond. Res..

[5] Senefeld J, Smith C, Hunter SK (2016). Sex differences in participation, performance, and age of ultramarathon runners. Int. J. Sports Physiol. Perform..

[6] Gloor RU, Knechtle B, Knechtle P, Rüst CA, Haupt S, Rosemann T, Lepers R (2013). Sex-related trends in participation and performance in the 'Swiss Bike Masters' from 1994–2012. Percept. Mot. Skills.

[7] Baumgartner S, Sousa CV, Nikolaidis PT, Knechtle B (2020). Can the performance gap between women and men be reduced in ultra-cycling?. Int. J. Environ. Res. Public Health.

[8] Nikolaidis PT, Villiger E, Knechtle B (2021). Participation and performance trends in the ITU Duathlon World Championship from 2003 to 2017. J. Strength Cond. Res..

[9] Romero-Ramos O, Fernández-Rodríguez E, Mayorga-Vega D, Merino-Marbán R, Podstawski R (2020). Sex and age-related changes in performance in the Duathlon World Championships. Rev. Bras. Med. Esporte.

[10] Rüst CA, Knechtle B, Knechtle P, Pfeifer S, Rosemann T, Lepers R, Senn O (2013). Gender difference and age-related changes in performance at the long-distance duathlon. J. Strength Cond. Res..

[11] World Triathlon. World Triathlon competition rules 2020 2019 URL: https://www.triathlon.org/uploads/docs/World_Triathlon_Sport_Competition_Rules_2020_201811253.pdf [accessed 2021–11–07].

[12] Nikolaidis PT, Villiger E, Ardigò LP, Waśkiewicz Z, Rosemann T, Knechtle B (2018). The age of peak performance in women and men duathletes: The paradigm of short and long versions in "Powerman Zofingen". Open Access J. Sports Med..

[13] Lepers R (2019). Sex difference in triathlon performance. Front. Physiol..

[14] Smith FW, Smith PA (2002). Musculoskeletal differences between males and females. Sports Med. Arthrosc. Rev..

[15] Tiller NB, Elliott-Sale KJ, Knechtle B, Wilson PB, Roberts JD, Millet GY (2021). Do sex differences in physiology confer a female advantage in ultra-endurance sport?. Sports Med..

[16] Waldvogel KJ, Nikolaidis PT, Di Gangi S, Rosemann T, Knechtle B (2019). Women reduce the performance difference to men with increasing age in ultra-marathon running. Int. J. Environ. Res. Public Health.

[17] Nikolaidis PT, Cuk I, Clemente-Suárez VJ, Villiger E, Knechtle B (2021). Number of finishers and performance of age group women and men in long-distance running: Comparison among 10 km, half-marathon and marathon races in Oslo. Res. Sports Med..

[18] Sousa-Victor P, García-Prat L, Serrano AL, Perdiguero E, Muñoz-Cánoves P (2015). Muscle stem cell aging: Regulation and rejuvenation. Trends Endocrinol. Metab..

[19] Lepers R, Stapley PJ, Cattagni T (2018). Variation of age-related changes in endurance performance between modes of locomotion in men: An analysis of master world records. Int. J. Sports Physiol. Perform..

[20] Allen SV, Hopkins WG (2015). Age of peak competitive performance of elite athletes: A systematic review. Sports Med..

[21] Bentley DJ, Millet GP, Vleck VE, McNaughton LR (2002). Specific aspects of contemporary triathlon: Implications for physiological analysis and performance. Sports Med..

[22] Millet GP, Vleck VE, Bentley DJ (2009). Physiological differences between cycling and running: Lessons from triathletes. Sports Med..

[23] Nikolaidis PT, Villiger E, Victor Sousa C, Rosemann T, Knechtle B (2019). The effect of aging on pacing strategies in short and long distance duathlon. Exp. Aging Res..

[24] Rüst CA, Knechtle B, Knechtle P, Rosemann T, Lepers R (2012). Age of peak performance in elite male and female Ironman triathletes competing in Ironman Switzerland, a qualifier for the Ironman world championship, Ironman Hawaii, from 1995 to 2011. Open Access J. Sports Med..

[25] Etter F, Knechtle B, Bukowski A, Rüst CA, Rosemann T, Lepers R (2013). Age and gender interactions in short distance triathlon performance. J. Sports Sci..

[26] Knechtle B, Rüst CA, Rosemann T, Lepers R (2012). Age and gender differences in half-Ironman triathlon performances: The Ironman 70.3 Switzerland from 2007 to 2010. Open Access J. Sports Med..

[27] World Triathlon. Results—World Triathlon 2023 URL: https://triathlon.org/results [accessed 2023-02-02].

[28] Internet Archive. Wayback Machine 2023 URL: https://web.archive.org/ [accessed 2023-02-02].

[29] Lamont M, Kennelly M, Wilson E (2012). Competing priorities as constraints in event travel careers. Tour. Manag..

[30] Scheer V (2019). Participation trends of ultra endurance events. Sports Med. Arthrosc. Rev..

[31] Thuany M, Gomes TN, Villiger E, Weiss K, Scheer V, Nikolaidis PT, Knechtle B (2022). Trends in participation, sex differences and age of peak performance in time-limited ultramarathon events: A secular analysis. Medicina (Kaunas).

[32] Shoak MA, Knechtle B, Knechtle P, Rüst CA, Rosemann T, Lepers R (2013). Participation and performance trends in ultracycling. Open Access J. Sports Med..

[33] Deutscher Olympischer Sportbund. Bestandserhebung 2022 URL: https://cdn.dosb.de/user_upload/www.dosb.de/uber_uns/Bestandserhebung/Bestandserhebung_2016.pdf [accessed 2023-14-05].

[34] Deutscher Olympischer Sportbund. Bestandserhebung 2001 URL: https://cdn.dosb.de/user_upload/www.dosb.de/uber_uns/Bestandserhebung/Bestandserhebung_2001.pdf [accessed 2023-14-05]

[35] Knechtle B, Tanous DR, Wirnitzer G, Leitzmann C, Rosemann T, Scheer V, Wirnitzer K (2021). Training and racing behavior of recreational runners by race distance-results from the NURMI study (step 1). Front. Physiol..

[36] Hallam LC, Amorim FT (2022). Expanding the gap: An updated look into sex differences in running performance. Front. Physiol..

[37] Bassett DR, Howley ET (2000). Limiting factors for maximum oxygen uptake and determinants of endurance performance. Med. Sci. Sports Exerc..

[38] Knechtle B, Valeri F, Nikolaidis PT, Zingg MA, Rosemann T, Rüst CA (2016). Do women reduce the gap to men in ultra-marathon running?. Springerplus.

[39] Gallmann D, Knechtle B, Rüst CA, Rosemann T, Lepers R (2013). Elite triathletes in ‘Ironman Hawaii’ get older but faster. Age (Dordr).

[40] Reusser M, Sousa CV, Villiger E, Alvero Cruz JR, Hill L, Rosemann T, Nikolaidis PT, Knechtle B (2021). Increased participation and decreased performance in recreational master athletes in "Berlin Marathon" 1974–2019. Front. Physiol..

[41] Maffetone PB, Malcata R, Rivera I, Laursen PB (2017). The Boston marathon versus the world marathon majors. PLoS ONE.

[42] Knechtle B, Di Gangi S, Rüst CA, Nikolaidis PT (2020). Performance differences between the sexes in the Boston marathon from 1972 to 2017. J. Strength Cond. Res..

[43] Vitti A, Nikolaidis PT, Villiger E, Onywera V, Knechtle B (2020). The, "New York City Marathon": Participation and performance trends of 1.2M runners during half-century. Res. Sports Med..

[44] Lepers R, Stapley PJ (2011). Age-related changes in conventional road versus off-road triathlon performance. Eur. J. Appl. Physiol..

[45] Bernard T, Sultana F, Lepers R, Hausswirth C, Brisswalter J (2009). Age-related decline in Olympic triathlon performance: Effect of locomotion mode. Exp. Aging Res..

[46] Bijker KE, de Groot G, Hollander AP (2002). Differences in leg muscle activity during running and cycling in humans. Eur. J. Appl. Physiol..

[47] Heiden T, Burnett A (2003). The effect of cycling on muscle activation in the running leg of an Olympic distance triathlon. Sports Biomech..

[48] Swinnen W, Kipp S, Kram R (2018). Comparison of running and cycling economy in runners, cyclists, and triathletes. Eur. J. Appl. Physiol..

[49] Knechtle B, Wirth A, Rosemann T (2010). Predictors of race time in male Ironman triathletes: Physical characteristics, training, or prerace experience?. Percept. Mot. Skills.

[50] Nikolaidis PT, Villiger E, Vancini RL, Rosemann T, Knechtle B (2018). The effect of sex and performance level on pacing in duathlon. Sports (Basel).

[51] Deaner RO, Addona V, Carter RE, Joyner MJ, Hunter SK (2016). Fast men slow more than fast women in a 10 kilometer road race. PeerJ.

[52] Knechtle B, Nikolaidis PT (2016). Sex differences in pacing during 'Ultraman Hawaii'. PeerJ.

[53] Deaner RO, Lowen A (2016). Males and females pace differently in high school cross-country races. J. Strength Cond. Res..

[54] Renfree A, do Carmo EC, Martin L (2016). The influence of performance level, age and gender on pacing strategy during a 100-km ultramarathon. Eur. J. Sport Sci..

[55] Nikolaidis PT, Chtourou H, Ramirez-Campillo R, Villiger E, Rosemann T, Knechtle B (2019). The combined effect of aging and performance level on pacing in duathlon: The "ITU Powerman Long Distance Duathlon World Championships". Front. Psychol..

